# Association between RMTg Neuropeptide Genes and Negative Effect during Alcohol Withdrawal in Mice

**DOI:** 10.3390/ijms25052933

**Published:** 2024-03-02

**Authors:** Yixin Fu, Wenfu Li, Yunlin Mai, Junhao Guan, Ruxuan Ding, Jiawei Hou, Bingqing Chen, Guoxin Cao, Shizhu Sun, Ying Tang, Rao Fu

**Affiliations:** 1 Department of Anatomy, School of Medicine, Sun Yat-Sen University, Shenzhen 518106, China; fuyx37@mail2.sysu.edu.cn (Y.F.); liwf35@mail2.sysu.edu.cn (W.L.); maiylin@mail2.sysu.edu.cn (Y.M.); guanjh25@mail2.sysu.edu.cn (J.G.); dingrx3@mail2.sysu.edu.cn (R.D.); houjw9@mail2.sysu.edu.cn (J.H.); chenbq29@mail2.sysu.edu.cn (B.C.); caogx3@mail2.sysu.edu.cn (G.C.); sunshzh5@mail.sysu.edu.cn (S.S.); 2 Clinical Skills Training Center, School of Medicine, Sun Yat-Sen University, Shenzhen 518106, China

**Keywords:** alcohol, RMTg, neuropeptides, anxiety, depression

## Abstract

Alcohol use disorders (AUDs) frequently co-occur with negative mood disorders, such as anxiety and depression, exacerbating relapse through dopaminergic dysfunction. Stress-related neuropeptides play a crucial role in AUD pathophysiology by modulating dopamine (DA) function. The rostromedial tegmental nucleus (RMTg), which inhibits midbrain dopamine neurons and signals aversion, has been shown to increase ethanol consumption and negative emotional states during abstinence. Despite some stress-related neuropeptides acting through the RMTg to affect addiction behaviors, their specific roles in alcohol-induced contexts remain underexplored. This study utilized an intermittent voluntary drinking model in mice to induce negative effect behavior 24 h into ethanol (EtOH) abstinence (post-EtOH). It examined changes in pro-stress (*Pnoc*, *Oxt*, *Npy*) and anti-stress (*Crf*, *Pomc*, *Avp*, *Orx*, *Pdyn*) neuropeptide-coding genes and analyzed their correlations with aversive behaviors. We observed that adult male C57BL/6J mice displayed evident anxiety, anhedonia, and depression-like symptoms at 24 h post-EtOH. The laser-capture microdissection technique, coupled with or without retrograde tracing, was used to harvest total ventral tegmental area (VTA)-projecting neurons or the intact RMTg area. The findings revealed that post-EtOH consistently reduced *Pnoc* and *Orx* levels while elevating *Crf* levels in these neuronal populations. Notably, RMTg *Pnoc* and *Npy* levels counteracted ethanol consumption and depression severity, while *Crf* levels were indicative of the mice’s anxiety levels. Together, these results underscore the potential role of stress-related neuropeptides in the RMTg in regulating the negative emotions related to AUDs, offering novel insights for future research.

## 1. Introduction

Alcohol use disorder (AUD) is characterized by loss of control over alcohol intake, compulsive alcohol use, and a negative emotional state when not drinking, which can follow a chronic, relapsing course [1,2,3]. AUD is also one of the most common mental illnesses globally since alcohol dependence and alcohol abuse will lead to substantial morbidity and mortality [4]. Thus, it is urgent to develop an effective therapeutic intervention for AUD, which requires a comprehensive understanding of the mechanisms underpinning neuroadaptations observed with alcohol-related affective disorder comorbidity.

Neuropharmacological research has well documented that the brain reward and stress-related circuitry become dysregulated with the onset of alcohol dependence [5], including alterations involving behavioral, physiological, and neurological events [6]. Extensive studies address the great potential of neuropeptide systems for AUD therapeutics, like the application of naltrexone [7,8], strongly suggest the importance of neuropeptides in the etiology of alcohol addiction development [9].

Neuropeptides, functionally classified as pro-stress (corticotropin-releasing factor, dynorphin, prepropimelanocortin, vasopressin, and orexin) and anti-stress (pro-nociceptin, oxytocin, and neuropeptide Y), have impacts on stress balance, negative emotional, and motivational consequences associated with alcohol abuse and dependence [6,10,11,12]. These stress-related neuropeptides also interact with neurotransmission in the brain [13].

The neuropeptides are stored and, by demand, secreted via regulated secretory pathways and bind to specific G-protein coupled receptors (GPCRs) widely distributed in the brain. Activation of these GPCRs triggers an intracellular cascade of molecular enzymatic events that result in cellular responses, initiating the transcription or controlling cellular excitability, modulating their firing patterns, and leading to transmitter release [14]. However, diverse and complicated molecular mechanisms are connected with stress-related neuropeptides during their process of modulating negative mood disorders. For example, oxytocin, regarded as one of the anti-stress neuropeptides, could improve depression-like behavior through hippocampal CREB-BDNF signaling [15]. Moreover, the reduced inflammatory cytokine (TNF-α, IL-6, and IL-1β) levels in the mPFc are also linked to its potential therapeutic effect against recognition and social interaction impairments [16]. Similarly, the anxiolytic effect of NPY involves suppression of the anxiety-associated genes (*Orx* and *Cck*) and catecholamine production (gr, mr, th1, and th2) in the brain [17]. Conversely, as one of the pro-stress neuropeptides, the CRF activates the HPA axis and subsequent glucocorticoid release in stress events [18,19,20]. It also promotes stress by modulating the midbrain dopamine system. It was reported that CRF activates VTA dopamine neurons via NMDA receptor potentiation, linked with CRF2 receptor/PLC/PKC cascade [19]. Orexin and its receptor, OX1R, were proposed to be positively correlated to depression and anxiety, which would be due to disinhibition from defective aminergic neurons [21]. However, OX2R has anti-depressive properties. The enhanced Ca^2+^ responses through canonical OX1R (or OX2R) activated PLC-IP3/DAG signaling or by OX1R mediated the transient receptor potential channel 3 pathway, and both might contribute to the anxiogenic and depressogenic effect of orexin [22]. Considering the midbrain dopamine, the tonic controlled by the RMTg serves as a critical neural substrate underlying drug abuse and mood regulation [23], during which neuropeptides participate. However, whether RMTg GABAergic neurons, particularly those projecting to the VTA, express mRNA encoding stress-related neuropeptides is less clear.

The rostromedial tegmental nucleus (RMTg), also known as the tail of ventral tegmental area (tVTA), has become a hotspot in the addiction research field due to its physiological function as a GABAergic master brake for dopamine systems [24,25,26]. Recently, RMTg hyperactivation was proposed to induce VTA DA hypofunction and its linked affective disorders during alcohol withdrawal [24,27,28].

The lack of clear delineation and cytoarchitectural boundaries with neighboring tissue provides a technique challenge for precisely isolating the RMTg tissue. This drawback partly accounts for a relatively slow advance in understanding the molecule mechanism involved in the behavioral function participated by RMTg. A recent study explored the gene expression and neurochemical characterization in rodent RMTg and proposed the *Pnoc* and *Foxp1* as selective markers, distinguishing the RMTg neuron from neighboring regions, like VTA [24,29]. However, we still have a knowledge gap regarding the function of stress-related neuropeptides in the RMTg for alcohol-related abnormal behavior.

Here, we trained male C57BL/6J mice to access alcohol within the intermittent access two-bottle-choice (IA2BC) drinking paradigm, which is a well-established rodent model that mimics clinically relevant aspects of human addiction [30]. By using the laser-capture microdissection (LCM) [24] coupled with VTA retrograde tracing techniques, we could isolate RMTg neurons with specific projections. We investigate the expression of stress-related neuropeptides encoding genes (including *Pnoc*, *Oxt*, *Npy*, *Crf*, *Pomc*, *Avp*, *Orx*, and *Pdyn*) and their correlation with the affective disorder associated with chronic alcohol exposure and withdrawal in mice. We reported here that the RMTg *Pnoc* and *Npy* expression counteracts ethanol consumption level and depression severity, while *Crf* represents anxiety severity.

## 2. Results

### 2.1. Expression of Anxiety- and Depression-like Behaviors after Alcohol Withdrawal

**Figure 1:**
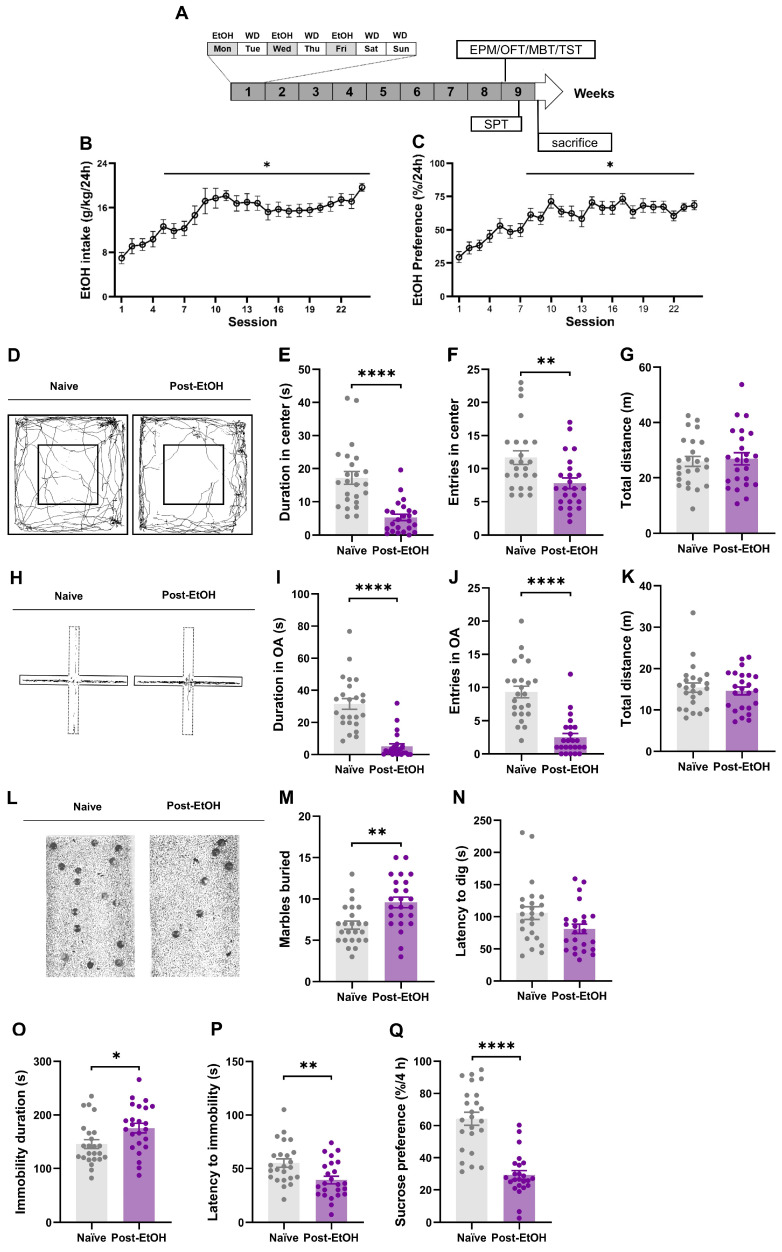
Mice subjected to chronic alcohol drinking and acute withdrawal exhibit anxiety- and depression-like behavior. (**A**) The schematic diagram shows that the male C57BL/6J mice consume 20% (*v*/*v*) ethanol (EtOH) within the intermittent access two-bottle choice (IA2BC) paradigm and negative effect behavior assessment. (**B**,**C**). EtOH intake and preference demonstrate significant elevation and stability at the eighth and ninth drinking session versus in the first session. (**D**) Typical trajectories of mice at 24 h into EtOH abstinence (post-EtOH) and their counterparts (naïve) in the open field test (OFT); (**E**–**G**) duration in the central area (s), number of times entering the central area, and total distance of movement in the OFT; (**H**) typical traces of mice in an elevated plus maze (EPM) test; (**I**–**K**) the duration of open arms (s), number of times entering the open arm (%), and the total distance in open and closed arms during the EPM test; (**L**) representative photographs of mice in the marble-burying test (MBT); (**M**,**N**) latency to dig and number of marbles buried in the MBT; (**O**,**P**) the immobility time (s) and the latency to immobility (s) during the tail suspension test (TST); (**Q**) the sucrose preference at 4 and 24 h (%). One-way RM ANOVA followed by Bonferroni post hoc test. * *p* < 0.05, ** *p* < 0.01, **** *p* < 0.0001 vs. naïve, unpaired Student’s *t*-test. n = 24 mice/group, each dot represents one individual mouse. All data are presented as mean ±  SEM.

The schematic in Figure 1A depicts the experimental design. The male C57BL/6J mice (n = 24) consumed 20% alcohol in an 8-week IA2BC paradigm. The control group is another group of mice drinking water in the same paradigm (naïve, n = 24). Specifically, mice had access to alcohol/water on Monday, Wednesday, and Friday and access to water on the rest of the days during the week. The choice of ethanol exposure time was based on previous studies showing that a long-term voluntary drinking history is sufficient to induce excessive ethanol intake [31]. Here, we observed these mice gradually and significantly escalated their ethanol intake and preference, reaching 19.74  ±  1.352 g/kg/24 h and 61.44 ± 7.482%, at the 24th session by the end of 8 weeks of training (Figure 1B,C).

Anxiety symptoms are often observed after prolonged alcohol abuse, particularly during acute withdrawal, which promotes relapse [24]. We assessed the anxiety-like behaviors using the elevated plus maze (EPM) test, open field test (OFT), and marble-burying test (MBT) in naïve and post-EtOH mice. We observed that post-EtOH mice displayed higher anxiety levels than their naïve counterparts. Statistical analysis revealed the significance of the duration in the center (Figure 1E, t = 3.614, *p* = 0.0023) and entries to the center (Figure 1F, t = 4.701, *p* = 0.0002) of the OFT, time spent in open arms (OA) (Figure 1I, t = 4.884, *p* = 0.0002), and entries to OA of the EPM (Figure 1J, t = 3.781, *p* = 0.0016), as well as latency to dig (Figure 1M, t = 5.243, *p* < 0.0001) and the number of marbles buried in the MBT (Figure 1N, t = 5.155, *p* < 0.0001) between groups.

Depression is common among people who abuse alcohol. Anhedonia and depression contribute significantly to relapse [32]. We assessed the depression-like behaviors using the tail suspension test (TST) and sucrose preference test (SPT) in naïve and post-EtOH mice. Statistical analysis revealed significance in the duration of immobility (Figure 1O, t = 2.759, *p* = 0.0140), latency to immobility (Figure 1P, t = 3.546, *p* = 0.0027) in the TST, as well as the sucrose preference (Figure 1Q, t = 3.602, *p* = 0.0024) in SPT. These behavioral variations suggested that post-EtOH mice showed despair and anhedonia symptoms, consistent with what we recently reported in rats [24].

### 2.2. Chronic Alcohol Consumption and Withdrawal Impair the Gene Expression of Stress-Related Neuropeptides in the RMTg

**Figure 2:**
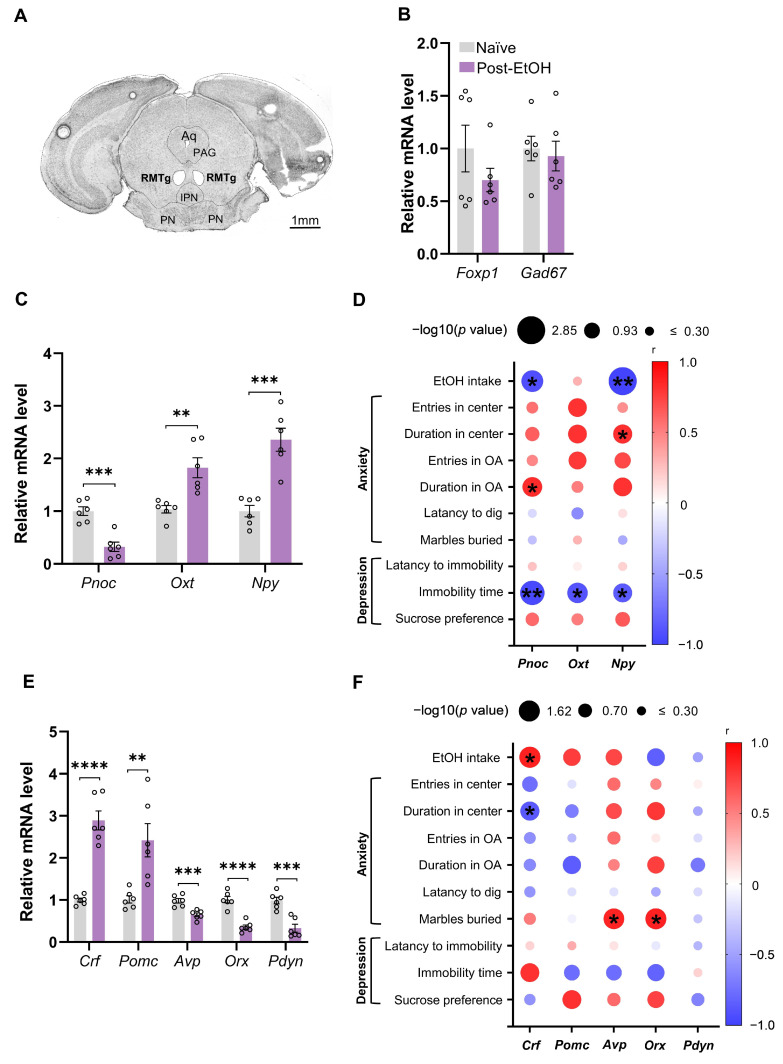
Expression of neuropeptide mRNA in RMTg of naïve and withdrawal mice. (**A**) Typical tissue map obtained by LCM, with the RMTg cavity showing the site of brain cutting regions; (**B**) RMTg *Foxp1*, *Gad67* relative expression levels between naïve and withdrawal mice; (**C**) RMTg anti-stress neuropeptide relative expression levels; (**D**) correlation analysis of RMTg anti-stress neuropeptide expression level with drinking, anxiety-, and depression-like behavior after alcohol withdrawal; (**E**) RMTg pro-stress neuropeptide relative expression levels; (**F**) correlation analysis of RMTg pro-stress neuropeptide expression level with drinking, anxiety-, and depression-like behavior after alcohol withdrawal. All data are presented as mean ±  SEM. * *p* < 0.05, ** *p* < 0.01, *** *p* < 0.001, **** *p* < 0.0001 vs. naïve, unpaired Student’s *t*-test, Pearson’s correlation coefficient analysis. For panels (**C**,**E**), each circle indicates a mean gene expression value of pooled RMTg tissue derived from two animals.

We collected RMTg tissues in post-EtOH and naïve mice using LCM for the gene expression test. Figure 2A depicts representative images of the coronal brain section with the RMTg dissected. The gene expression of *Foxp1* and *Gad67*, markers of RMTg GABAergic neurons, were examined to validate the accuracy of tissue harvesting by the LCM technique. The results showed no significant alteration between groups (Figure 2B).

Within the anti-stress neuropeptides [33], the qPCR result showed that post-EtOH mice had significantly reduced *Pnoc* and enhanced *Oxt* and *Npy* mRNA levels in the RMTg compared to the naïve group (Figure 2C, all *p* < 0.05). Moreover, within the pro-stress neuropeptides, post-EtOH mice presented substantially elevated *Crf* and *Pomc* but decreased *Avp*, *Orx*, and *Pdyn* mRNA levels in the RMTg compared to naïve counterparts (Figure 2E, all *p* < 0.05).

As illustrated in Figure 2D,F, the correlation analysis revealed that EtOH intake has significant negative correlations to anti-stress neuropeptides *Pnoc* and *Npy* mRNA expression. In contrast, it is positively correlated to the pro-stress neuropeptide *Crf* expression in the RMTg of post-EtOH mice.

Additionally, despair behavioral measures had significant negative correlations to the mRNA level of genes encoding the anti- but not pro-stress neuropeptides in the RMTg. Notably, TST immobility time was significantly negatively correlated to RMTg *Pnoc*, *Oxt*, and *Npy* gene expression of post-EtOH mice.

Also, significant positive correlations occurred between anxiolytic behavioral measures (duration in center/open arm) and anti-stress neuropeptide gene levels (*Pnoc*, *Npy*). They also existed between anxiogenic behavioral measures (number of marbles buried) and pro-stress neuropeptide genes (*Avp*, *Orx*). Similarly, negative correlations occurred between anxiolytic behavioral measures and pro-stress neuropeptide genes (*Crf*).

All changes in the mRNA expression of genes and their correlation with behavior are summarized in Table 1. These findings suggest that chronic alcohol exposure and withdrawal influence the balancing act of pro- and anti-stress neuropeptide gene expression in the RMTg.

### 2.3. Chronic Alcohol Consumption and Withdrawal Impair the Gene Expression of Stress-Related Neuropeptides in the VTA-Projecting RMTg Neurons

**Figure 3:**
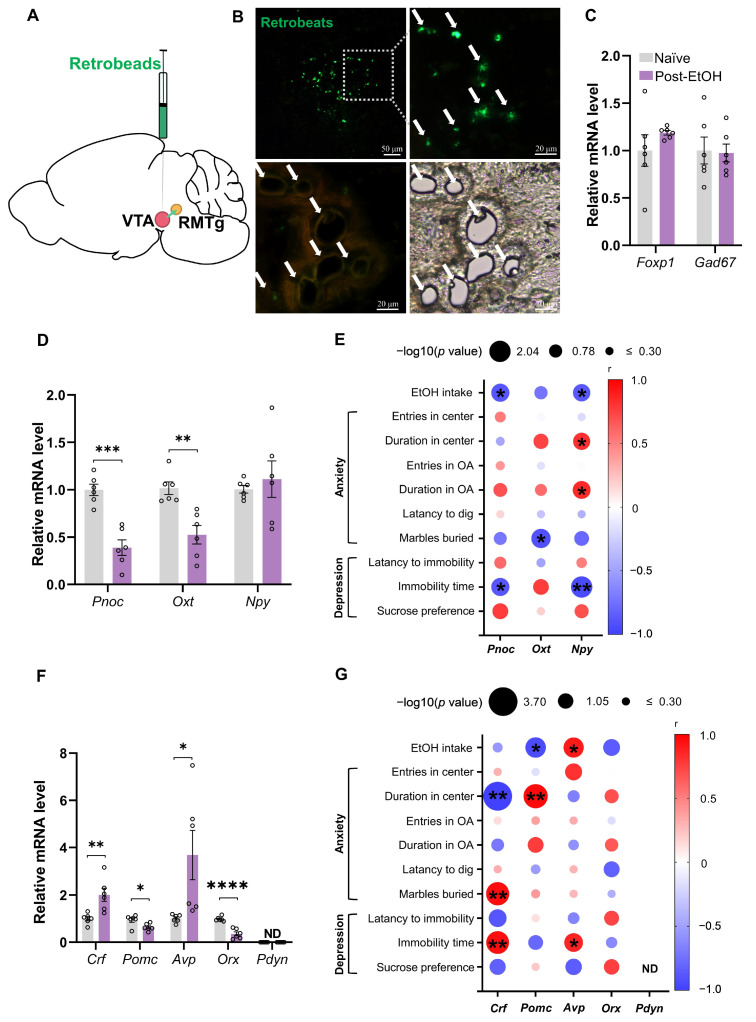
Expression of neuropeptide mRNA in VTA-projecting RMTg neurons of naïve and withdrawal mice. (**A**) Green retrograde tracer injection schematic diagram, the injection site in VTA, and the return site in RMTg. (**B**) Typical tissue map obtained by LCM, with a small cavity showing the location of cutting single cells. Each white arrow represents a VTA-projecting RMTg cell; (**C**) VTA-projecting RMTg specific cells *Foxp1*, *Gad67* relative expression levels between naïve and withdrawal mice; (**D**) VTA-projecting RMTg cell anti-stress neuropeptide relative expression levels; (**E**) correlation analysis of VTA-projecting RMTg cell anti-stress neuropeptide expression level with drinking, anxiety-, and depression-like behavior after alcohol withdrawal; (**F**) VTA-projecting RMTg cell pro-stress neuropeptide relative expression levels; (**G**) correlation analysis of VTA-projecting RMTg cell pro-stress neuropeptide expression level with drinking, anxiety-, and depression-like behavior after alcohol withdrawal. All data are presented as mean ±  SEM. * *p* < 0.05, ** *p* < 0.01, *** *p* < 0.001, **** *p* < 0.0001 vs. naïve, unpaired Student’s *t*-test, Pearson correlation coefficient analysis. ND: no detection. For panels (**D**,**F**), the individual point indicates a mean gene expression value of pooled RMTg tissue derived from two animals.

Next, we combined the VTA retrograde neurotracing and qPCR to examine how chronic alcohol exposure and withdrawal alter neuropeptide expression in VTA-projecting RMTg neurons in post-EtOH and naïve mice (Figure 3A).

We provided histological evidence showing that the retrobeads are concentrated in the RMTg region of mice that received intra-VTA Green IX retrobead microinfusion for 9–11 days. Individual VTA-projecting neurons could be identified with green fluorescence and captured using LCM (Figure 3B).

The qPCR result revealed that *Foxp1* and *Gad67* were examined in the individual projecting neurons, validating the RMTg molecular characterization, although they were not significantly altered post-EtOH (Figure 3C).

Compared to the naïve group, the qPCR result showed that post-EtOH mice had significantly reduced *Pnoc* and *Oxt* in the VTA-projecting RMTg neurons among the anti-stress neuropeptides (Figure 3D, both *p* < 0.05). Moreover, post-EtOH mice exhibited augmented *Crf* and *Avp* but decreased *Pomc* and *Orx* mRNA levels among the pro-stress neuropeptides (Figure 3F, all *p* < 0.05). It is noted that *Pdyn* mRNA expression was not detected in the VTA-projecting RMTg neurons of mice.

Next, we investigated the relationship between neuropeptides within VTA-projecting RMTg neurons and ethanol-related behaviors (Figure 3E,G). Firstly, the EtOH consumption negatively correlated to anti-stress neuropeptides *Pnoc* and *Npy* but positively correlated to the pro-stress *Avp* level. Similarly, the severity of despair in the TST negatively correlated to anti-stress neuropeptides *Pnoc* and *Npy* but positively correlated to the *Crf* and *Avp* levels. Moreover, the anxiety degree during behavioral tests negatively correlated to anti-stress neuropeptides *Oxt* and *Npy* but positively correlated to pro-stressed *Crf* level.

The complete correlation analysis results between the gene expression and each behavioral measure are listed in Table 2. These findings indicate that chronic alcohol exposure and repeated abstinence may cause stress-related neuropeptide change in the individual VTA-projecting RMTg neurons.

## 3. Discussion

In this study, we observed that mice subjected to chronic intermittent alcohol consumption exhibited stable patterns of alcohol intake, accompanied by symptoms of anxiety and depression during withdrawal periods. Moreover, post-EtOH mice consistently exhibited reduced *Pnoc* and *Orx* and elevated *Crf* expression level in both VTA-projecting RMTg neurons and the RMTg region as a whole. Despite alterations in the expression of genes such as *Oxt*, *Npy*, *Pomc*, *Avp*, and *Pdyn* in the RMTg, which are implicated in stress regulation, these genes exhibited diverse and inconsistent expression patterns in VTA-projecting neurons. Notably, *Pnoc* and *Npy* expression demonstrated an inverse association with ethanol consumption and the severity of depressive symptoms, whereas *Crf* expression paralleled the intensity of anxiety levels.

Exploring AUD’s neurobiology improves treatments for patients with co-occurring mood disorders. The Fawn-Hooded rat model has unveiled key behavioral and neurobiological insights [34]. We previously used various alcohol exposure methods (e.g., voluntary drinking, operant self-administration, and vapor inhalation) in rats, which have elucidated the reduced function of brain monoaminergic systems due to overactive anti-reward circuits like LHb and RMTg, contributing to the development of a negative effect in AUD [24,27,35,36,37,38,39,40]. While rats offer insights into AUD and its comorbid negative effect, mice provide superior advantages due to the extensive range of transgenic and inbred strains and the ease of generating specific genetic mutations or manipulating neural circuits [41,42,43]. Therefore, transitioning studies from rats to mice is crucial for advancing our understanding of the genetic and neural underpinnings of AUD and negative mood comorbidity.

We trained male C57BL/6J mice to consume 20% ethanol for eight weeks using the IA2BC paradigm, leading to a gradual increase in voluntary ethanol intake, preference, and pharmacologically relevant blood ethanol concentrations (BECs). After seven sessions (2–3 weeks), mice reached excessive drinking levels (18–21 g/kg/24 h) and maintained this, aligning with a previous finding [44]. Though BECs were not measured here, past studies noted BECs exceeding 100 mg/dL after binge drinking [41,45]. Chronic voluntary drinking in mice resulted in behaviors indicative of a negative effect during acute withdrawal, consistent with prior research [44,46]. These animal behaviors mirror symptoms of anxiety and depression seen in patients with comorbid AUD, affirming the animal model’s face validity [47,48]. Both clinical and preclinical studies identify shared targets like mGluR5 for potential AUD treatments, underscoring the model’s validity [49,50]. Furthermore, the model exhibits construct validity, as voluntary drinking mice and AUD patients both show neurochemical irregularities, such as reduced hippocampal and nucleus accumbens volumes [51,52].

RMTg neurons encode negative reward signals and are excited by aversive stimuli [25,53,54]. RMTg GABAergic neurons, genetically distinct from VTA, exert a robust inhibitory drive onto midbrain dopamine neurons [55,56], serving as a strong “brake” on the dopaminergic system [26,57].

We recently reported that RMTg plays a role in modulating behaviors related to ethanol consumption. Our proposal suggests that abnormal hyperactivity in RMTg GABAergic neurons leads to the diminished activity of VTA DA neurons, which is linked to the co-occurrence of anxiety and depression symptoms during alcohol withdrawal [24,28].

However, the RMTg functional research, particularly in neuropeptides and neuromodulators, lags behind VTA studies due to challenges in distinctively defining RMTg’s boundaries [29]. Addressing this, we recently used LCM and qPCR to isolate RMTg from frozen tissue precisely, investigating N/OFQ-NOP signaling changes in rats post-chronic alcohol exposure and withdrawal [24].

Laser capture microdissection (LCM) enables precise access to brain regions using anatomical markers for identification [58]. To ensure accurate dissection and high-quality RNA, our protocol included rapidly freezing the brain in liquid nitrogen then sectioning it into 30 μm thick coronal slices with a cryostat based on stereotaxic coordinates. Sections were quickly counterstained with Nissl stain to aid in morphology identification before LCM.

Neuropeptides modulate stress, impacting anxiety and alcohol-related behaviors, with alcohol use making individuals more susceptible to stress—a key factor in alcohol seeking and relapse [59,60,61]. We investigated genes for neuropeptides involved in mood disorders and AUD [33]. Compared to naïve mice, post-EtOH mice exhibited decreased *Pnoc* and enhanced *Oxt* and *Npy* among anti-stress neuropeptides as well as decreased *Orx*, *Pdyn*, and *Avp* but elevated *Pomc* and *Crf* in the RMTg among stress neuropeptides. Nevertheless, post-EtOH mice showed decreased *Oxt* and *Pomc*, but augmented *Avp* levels were observed in VTA-projecting RMTg neurons.

For both intact RMTg and VTA-projecting neurons, we observed consistently decreased *Pnoc*. This result generally aligns with our previous observation in a rat study [24]. In addition, *Pnoc* level negatively correlated to alcohol consumption amount, anxiety, and depression severity, indicating a potential anxiolytic and anti-depressant effect in the RMTg in response to alcohol exposure.

*Pnoc* encodes prepronociceptin, the precursor protein of N/OFQ, serving as the brain’s stress buffer system [24]. We proposed that the mice with RMTg N/OFQ insufficiency may drive the negative mood and excessive drinking behavioral pattern. This assumption is well supported by our finding that intra-RMTg N/OFQ infusion significantly reduces alcohol consumption in rats [24]. Furthermore, studies reported that selective N/OFQ system functional impairment contributes to excessive alcohol consumption and anxiety-like behavior [62,63], strongly backing the current assumption.

The neuropeptide Y (NPY) system, which is densely expressed in regions related to reward and emotion [64], is a potential therapeutic target for AUDs [60,65]. We observed an elevated *Npy* mRNA level in the RMTg, negatively correlated to ethanol consumption. This observation is consistent with reports that high-alcohol-consuming rodent genetic lines express lower brain levels of NPY, while low-consuming lines show higher NPY expression [66,67,68]. The NPY, derived from the hypothalamus, inhibits VTA GABAergic neurons, disinhibiting the DA neuron [69], which underlies the previous observation that innate NPY levels might counteract ethanol-drinking behavior [70]. We propose that the NPY targeting RMTg GABAergic neurons may share this mechanism for modulating drinking behavior.

Oxytocin affects cognitive and emotional behavior, exerts stress-buffering and prosocial roles [71] in the brain, and has therapeutic potency in AUD patients [72]. These effects would be mediated by the widely distributed oxytocin receptor (OTR) in the brain [73]. Here, the RMTg *Oxt* expression negatively correlated to the depression state of post-EtOH mice, keeping the general function of oxytocin consistent.

However, their expression appeared distinct between RMTg and VTA-projecting RMTg neurons, suggesting a potentially complicated role of oxytocin in this region. Indeed, within the midbrain, besides the dopaminergic and glutamatergic neurons, OTR is expressed in the GABAergic interneurons within the VTA [73], making the modulatory role of oxytocin on the DA-associated function complex.

Given that input-specific control of reward and aversion in the ventral tegmental area is a distinct function [74], we propose that the working mechanisms underlying the oxytocin modulating the DA system involve the direct, or even indirect, excitation and inhibition by disinhibition of DA neurons via RMTg. Since the change of alcohol withdrawal in the oxytocin is region-specific [72], it is likely that the enhanced oxytocin-OTR signaling on the RMTg leads to a relatively low monoaminergic level during alcohol withdrawal.

CRF, a 41-amino-acid neuropeptide found throughout the mammalian brain, activates the HPA axis and triggers glucocorticoid release during stress [18,19,20]. Elevated *Crf* expression in RMTg and VTA-projecting RMTg neurons during withdrawal suggests its role in disrupting the monoaminergic system, including dopamine, contributing to low mood states during stressful abstinence. We propose that the LHb-RMTg-VTA neural circuitry is the neural substrate underlying the involvement of CRF activity in mood disorder regulation changed by chronic alcohol exposure, independent of the HPA axis [11]. LHb, a primary excitatory input to the RMTg, is also hyperactive during alcohol withdrawal [24,37,75], partly accounting for RMTg neuronal abnormal hyperactivation [24,28].

Since the CRF could potentiate the NMDA receptor-mediated EPSCs via a presynaptic effect on the glutamate release, a post-synaptic modification on the NMDA receptor, or both [19,20], future study will examine the impact of CRF signaling on LHb-RMTg circuits. We assume the effect of CRF antagonists on excessive drinking reduction in dependent animals [18,76] may be working through the restored excitatory inputs to the RMTg and normalized neuronal activation, consequently rescuing related mood disorders.

Orexinergic neurons in the hypothalamus play key roles in regulating feeding, arousal, sleep–wake cycles, stress, and alcohol-related behavior [77]. Acute stress elevates orexin mRNA levels and activates orexin neurons, with orexin receptor antagonism having anxiolytic effects. However, chronic stress impacts the orexin system in complex and variable ways, influenced by the stressor type and brain region [78]. The orexin system is thought to influence alcohol’s reinforcing effects, particularly within the mesolimbic system (e.g., VTA, NAc), where blocking the orexin type 1 receptor notably decreases alcohol consumption [79].

We found that *Orx* levels in VTA-projecting RMTg neurons decreased and were positively correlated with anxiety severity in post-EtOH mice. Recent neurotracing identified an anatomical link between lateral hypothalamus orexinergic neurons and RMTg [79]. The presence of orexin receptors in the RMTg merits further study. We hypothesize that hyperactivity in the Orexin^LH^-GABA^RMTg^ circuit could contribute to the expression of negative mood-related behaviors in AUD.

Interestingly, the divergent trends of *Npy* and *Orx* suggest that an imbalance between these “anti-stress” and “pro-stress” peptides in the hypothalamus could affect the monoaminergic system and mood behavior related to AUD. The mechanism likely involves NPY and ORX from the hypothalamus modulating RMTg neuronal activity in a pre- or post-synaptic manner, a hypothesis currently under investigation in our ongoing research.

Gene expression variations were observed in the RMTg and its projections to the VTA post-ethanol exposure. *Pdyn*, encoding prodynorphin (a precursor to dynorphins that bind to κ-opioid receptors), is notably affected. Dynorphin, prevalent in brain regions linked to neuropsychiatric diseases like addiction and mood disorders [80], cyclically interacts with the ethanol system. Enhanced dynorphin transmission can increase ethanol consumption, while excessive drinking boosts dynorphin expression and its effects. The activation of the dynorphin/κ-opioid receptor system is tied to the negative reinforcement seen in alcohol addiction, particularly during acute withdrawal in rodents [81,82,83].

The decrease in *Pdyn* mRNA levels in the RMTg, influenced by interactions with other neuromodulatory or neuropeptide systems [84,85], suggests dynamic changes and possible compensatory mechanisms like CRF activation.

For example, CRF overexpression leads to increased PDYN protein in mice after morphine administration [86]. Moreover, while certain neuropeptides are linked to “pro-stress” responses and alcohol addiction, their variation during alcohol withdrawal is not uniform. Specifically, *Pdyn* mRNA levels decrease without significant changes in *Pomc* levels in alcohol-withdrawn rats [87], highlighting neuropeptides’ complex interplay and unique temporal and spatial responses during withdrawal.

Although the single-house method is crucial for monitoring alcohol intake in experiments [24], it is important to note its potential drawback. This method effectively isolates mice socially, exacerbating anxiety and depression, and affects neuropeptide levels (e.g., AVP, OXT), even in naïve mice [88,89,90]. While correlation analysis was used to link gene expression changes to behaviors in alcohol intake, anxiety, and depression-like behavior, it is important to note that pharmacological and behavioral experiments are needed to confirm the role of peptides in the RMTg.

## 4. Materials and Methods

### 4.1. Animals

Forty-eight male C57BL/6J mice (6–8 weeks at the start of the experiments) were obtained from the Laboratory Animal Center of Sun Yat-sen University and used in the investigation. All procedures were performed according to the Chinese National Health and Medical Research Council animal ethics guidelines and with the approval of the Sun Yat-sen University Animal Experimentation Ethics Committee (SYSK 2017-0081). Mice were housed individually in home cages in a climate-controlled room (20 °C) with a 12 h light/dark cycle. Animals were allowed to acclimate to the housing conditions and handling before the start of each experiment. Their body weight was recorded weekly. Food and water were available ad libitum. Animals were brought to the experiment room 30–40 min before each trial to let them habituate to an environment change.

### 4.2. Drugs

Alcohol drinking solutions were prepared from 95.0% alcohol solution (Yuecan, Guangzhou, China) and diluted into 20% solutions (*v*/*v*) using tap water. Sucrose (1%, *w*/*v*) was dissolved in tap water. Green IX Retrobeads^TM^ (Lumafluor Inc., Durham, NC, USA) were used for microinjection. No food or water deprivation was applied before or during the test.

### 4.3. Intermittent Access to 20% Ethanol in the Two-Bottle Free-Choice (IA2BC) Drinking Procedure

As reported, we trained mice to drink alcohol in the IA2BC paradigm for 8–12 weeks [31,91,92]. Starting Monday afternoon, mice were exposed to two bottles simultaneously for 24 h, one containing 20% ethanol (EtOH, *v*/*v*) and the other containing water. After 24 h, we replaced the ethanol bottle with a water bottle for the next 24 h. This pattern was repeated on Wednesday and Friday. On other days, mice could use two water bottles indefinitely. Alternate ethanol bottles were placed during each ethanol consumption process to control side effect preferences. The amount of ethanol or water consumed was determined by weighing the bottle before and after entering 24 h. Ethanol consumption was determined by calculating the grams of alcohol consumed per kilogram of body weight. The weekly “drip” average (fluid loss in cages without animals present) was subtracted from the individual fluid intake. Within 24 h, overflow was always less than 1.0 mL (less than 2.5% of the total liquid intake). The weight of all mice was recorded every week. In this mode, the ethanol intake and preference increased.

### 4.4. Open Field Test (OFT)

Each mouse was placed in the perimeter of the open field (50 × 50 × 50 cm), and their movements were recorded for 5 min, including center entries, duration in the center of the field (25 × 25 cm), and their overall distance traveled. The total movement distance of the mice was described as exercise ability, and the central zone time was used to assess anxiety behavior. The equipment was cleaned with a 10% ethanol solution to remove the fragrance of previously tested animals between tests [93,94]. A video tracking system (BAS-100, Tai Meng Technology, Chengdu, China) was used to record OFT and EPM behavior and for data analysis.

### 4.5. Elevated Plus Maze (EPM)

The elevated plus maze was raised 50 cm and made of black polypropylene. It comprised two arms (65 × 6 cm) that crossed over a center platform in a plus shape. The closed arms had a 20 cm translucent wall surrounding them, while the open arms had a 1cm border around their perimeter. The mouse was placed in the center of the apparatus facing the enclosed arm, and every 5 min session was videotaped. A 10% ethanol solution was used to clean the device between each animal test. The number of entries into the open arms and the time spent in the open arms were scored. The total number of entries into the arms (enclosed plus open) and the total distance were also calculated [95,96].

### 4.6. Marble-Burying Test (MBT)

This test used a white plexiglass cage (18 × 30 × 19 cm) with approximately 5 cm deep wood chip bedding lightly pressed to ensure a flat surface. Fifteen glass marbles were evenly spaced over the bedding. The mice were positioned in the center of the marble-containing cage, and throughout the 30 min test, the latency to dig was manually recorded for each animal. Then, the mouse was returned to its home cage, and the number of buried marbles was scored. A marble was partially buried if at least 70% of its surface was covered by bedding. Marbles were considered fully buried when no longer visible [97].

### 4.7. Tail Suspension Test (TST)

Mice were suspended by their tails for 6 min in the TST. We passed the tail through a lightweight plastic tube (0.5 g) to avoid tail climbing because C57BL6/J mice exhibit extensive tail-climbing activity. Mice were acoustically and visually isolated and held 50 cm above the ground by adhesive tape positioned 1 cm from the tip of the tail. The duration of immobility was measured during the latter 5 min span. The total time the tail suspension-induced immobility lasted and latency to immobility were measured [98,99].

### 4.8. Sucrose Preference Test (SPT)

The SPT, as described in this research, was used to examine depressive behavior [100]. Briefly, SPT was conducted 24 h after ethanol withdrawal. Before the test, mice were deprived of food and water for 20 h, after which they received a bottle of 1% sucrose solution and a bottle of water for 4 h. Sucrose intake was calculated based on the consumption of milligrams of sucrose per gram of body weight. The preference for sucrose was calculated based on the proportion of consumed sucrose solution in the overall amount of drinking liquid.

### 4.9. Stereotaxic Surgery and Microinjection Procedure

Mice were subjected to surgery using a stereotaxic apparatus (RWD Life Science, Shenzhen, China) while under anesthesia, induced by ketamine/xylazine (80/20 mg/kg, i.p.) and maintained with isoflurane as previously reported [24]. To label VTA-projecting RMTg neurons, we delivered Green Retrobeads IX^TM^ (200 nL/side) with a micropipette into the VTA (AP: −3.3 mm; ML: ± 1.35 mm; DV: −4.6 mm) of mice very slowly (5 nL/s) to minimize tissue damage. The micropipette was left in situ 10 min after injection to stop backflow. Then, sterile bone wax was used to seal the burr holes; mice were administered meloxicam (1.0 mg/kg, s.c.) before being put back in their home cage to recover from anesthesia. To achieve optimal tracer expression in the location, we sacrificed the mice 9–11 days following tracer injections.

### 4.10. Complete Laser Capture Microdissection Process

Based on the previously described method, we harvested RMTg brain tissue using an LCM 7 machine (Leica Microsystems, Wetzlar, Germany) for the molecular experiment. Briefly, isoflurane-induced profound anesthesia was used to decapitate mice. Brains were rapidly removed and placed in an optimal cutting temperature (OCT) medium (Sakura Fineteck, Torrance, CA, USA). The cryostat was used to cut the RMTg-containing brain tissue blocks into 30 μm coronal sections, which were then mounted on RNase-free, room-temperature polyethylene naphthalene (PEN) membrane slides (Leica Microsystems, Wetzlar, Germany). After a light cresyl violet staining, the slides proceeded immediately to LCM. Laser parameters were set up as follows: magnification 5×, power 56, aperture 9, speed 3, specimen balance 17, pulse frequency 501. The anatomical identification of the RMTg region was according to the earlier study [29,101], and tissue was collected, placed in a 0.5 mL Eppendorf tube, and then stored at −80 ℃ until RNA extraction.

### 4.11. RNA Extraction and Real-Time Quantitative PCR

Total RNA from LCM samples was extracted using the RNeasy micro kit (Catalog No. 74004, Qiagen Inc., Boston, MA, USA). RMTg tissue was resuspended in 30 μL lysis buffer and vortexed for 30 s, then treated following the instructions of the RNeasy Micro Kit for Microdissected Cryosections, and the total RNA was eluted with 10 μL of RNase-free water. RNA was quantified by a spectrophotometer Nanodrop One (Thermo scientific, Waltham, MA, USA) in absorbance ratios at 260–280 nm of 1.8–2.0. The cDNA was synthesized using the HiSlid ™ cDNA Synthesis Kit for qPCR (with dsDNase) (MIKXCo., Ltd., MKG840, Shenzhen, China). The primers were designed and obtained from Sangon Biotech. The quantitative PCR was performed on a QuantStudio™ 5 Real-Time PCR Detection System (Thermofisher, Waltham, MA, USA) using the 2 × Polarsignal^@^ qPCR mix (MIKXCo., Ltd., MKG800, Shenzhen, China) under the following conditions: 20 s at 94 °C followed by 45 cycles of 10 s at 94 °C and 20 s at 60 °C. Real-time PCR was performed in triplicate for each sample.

### 4.12. Statistical Methods

All data were expressed as a mean ± SEM (standard error of the mean). Animal sample sizes were determined to guarantee sufficient statistical power. Animals were allocated at random to several studies. Investigators blinded the group allocations in the behavioral trials. The behavioral tests, including drinking, anxiety-, and depression-like behaviors, between naïve and post-EtOH mice were analyzed using unpaired Student’s *t*-test analysis and one-way repeated-measures (RM) ANOVA followed by Bonferroni post-hoc tests. Statistical significance was declared at *p* < 0.05. Pearson’s correlation was used to analyze the correlation between mRNA levels and behavior tests.

## Figures and Tables

**Table 1:** The correlation of total RMTg neuropeptides’ mRNA expression with the behaviors of post-EtOH mice.

Total RMTg	Anti-Stress Neuropeptide mRNA Levels						Pro-Stress Neuropeptide mRNA Levels									
	*Pnoc*		*Oxt*		*Npy*		*Crf*		*Pomc*		*Avp*		*Orx*		*Pdyn*	
Behaviors	r	*p*	r	*p*	r	*p*	r	*p*	r	*p*	r	*p*	r	*p*	r	*p*
EtOH intake	−0.8855	0.0189	0.3069	0.5540	−0.9689	0.0014	0.8711	0.0238	0.7642	0.0769	0.7253	0.1029	−0.7784	0.0682	−0.4168	0.4111
Entries in center	0.5582	0.3116	0.8050	0.0534	0.4370	0.3862	−0.6882	0.1307	−0.1290	0.8075	0.5765	0.2310	0.4752	0.3409	0.0611	0.9084
Duration in center	0.6286	0.1813	0.8066	0.0525	0.8160	0.0477	−0.8177	0.0468	−0.6013	0.2067	0.7035	0.1188	0.7859	0.0638	−0.3824	0.4545
Entries in OA	0.4740	0.3422	0.7771	0.0690	0.7145	0.1106	−0.5067	0.3050	−0.2983	0.5658	0.5771	0.2304	0.0874	0.8693	−0.1422	0.7881
Duration in OA	0.8340	0.0390	0.5078	0.3037	0.8063	0.0526	−0.5295	0.2800	−0.7931	0.0598	0.4970	0.3159	0.7530	0.0840	−0.6402	0.1708
Latancy to dig	−0.1576	0.7656	−0.5225	0.2876	0.1247	0.8139	−0.4761	0.3398	−0.1350	0.7987	−0.1258	0.8123	−0.3784	0.4595	−0.1595	0.7628
Marbles buried	−0.2683	0.6072	0.2963	0.5685	−0.3715	0.4684	0.5186	0.2919	−0.0570	0.9145	0.8611	0.0276	0.8678	0.0251	−0.2506	0.6319
Latancy to immobility	0.2406	0.6460	0.0650	0.9026	0.1752	0.7400	0.1718	0.7449	0.3270	0.5271	0.1102	0.8354	−0.0964	0.8559	−0.3205	0.5357
Immobility time	−0.9316	0.0069	−0.8550	0.0300	−0.8134	0.0490	0.8101	0.0507	−0.6996	0.1218	−0.6946	0.1256	−0.7543	0.0832	0.1751	0.7400
Sucrose preference	0.5900	0.2177	0.5128	0.2982	0.6518	0.1607	−0.4827	0.3322	0.8105	0.0505	0.5854	0.2222	0.7429	0.0906	−0.5752	0.2323

**Table 2:** The correlation of VTA-projecting RMTg neuropeptides’ mRNA expression with the behaviors of post-EtOH mice.

VTA−Projecting RMTg	Anti-Stress Neuropeptide mRNA Levels						Pro-Stress Neuropeptide mRNA Levels							
	*Pnoc*		*Oxt*		*Npy*		*Crf*		*Pomc*		*Avp*		*Orx*	
Behaviors	r	*p*	r	*p*	r	*p*	r	*p*	r	*p*	r	*p*	r	*p*
EtOH intake	−0.8463	0.0336	−0.6530	0.1597	−0.8243	0.0436	−0.4623	0.3560	−0.9032	0.0136	0.8968	0.0154	−0.8050	0.0533
Entries in center	0.5180	0.2925	−0.0312	0.9532	−0.1481	0.7795	0.3088	0.5515	−0.1333	0.8013	0.8078	0.0519	0.0052	0.9922
Duration in center	−0.3873	0.4481	0.7353	0.0959	0.8134	0.0490	−0.9891	0.0002	0.9591	0.0025	−0.5840	0.2237	0.6864	0.1321
Entries in OA	0.4082	0.4218	−0.1236	0.8155	−0.0155	0.9768	0.1324	0.8026	0.3716	0.4682	0.3270	0.5270	−0.1397	0.7919
Duration in OA	0.6803	0.1370	0.5808	0.2268	0.8388	0.0369	−0.6253	0.1843	0.7641	0.0769	−0.5007	0.3117	0.6527	0.1600
Latancy to dig	0.1659	0.7534	−0.2498	0.6331	−0.3339	0.5178	0.3138	0.5448	−0.4438	0.3780	0.2948	0.5706	−0.7710	0.0726
Marbles buried	−0.6355	0.1750	−0.8786	0.0212	−0.7239	0.1038	0.9468	0.0042	0.4012	0.4304	0.2859	0.5828	−0.3468	0.5006
Latancy to immobility	0.5892	0.2185	−0.4074	0.4226	0.4999	0.3126	−0.8361	0.0381	0.1284	0.8084	−0.5644	0.2433	0.7258	0.1025
Immobility time	−0.8463	0.0336	0.7636	0.0772	−0.9209	0.0091	0.9420	0.0049	−0.7224	0.1049	0.8606	0.0278	−0.5411	0.2676
Sucrose preference	0.7685	0.0742	0.1918	0.7158	0.6888	0.1302	−0.7763	0.0694	0.2172	0.6794	−0.7885	0.0623	0.7496	0.0862

## Data Availability

The data presented in this study are available on request from the corresponding author.
